# Metformin attenuates diabetic osteoporosis via the miR-21 mediated Mef2c/Sost pathway

**DOI:** 10.3389/fendo.2026.1841140

**Published:** 2026-06-05

**Authors:** Le Liu, Jie Xu, Hui Zheng, Xiao-chen Li, Zhong-ai Gao, Cheng Meng, Congqing Pan, Ju-hong Yang, Bao-cheng Chang

**Affiliations:** 1 Department of Geriatrics, Second Hospital of Tianjin Medical University, Tianjin, China; 2 NHC Key Lab of Hormones and Development and Tianjin Key Lab of Metabolic Diseases, Tianjin Medical University Chu Hsien-I Memorial Hospital & Institute of Endocrinology, Tianjin, China; 3 Endocrinology Department, TEDA International Cardiovascular Hospital, Tianjin, China; 4 Department of Nephrology, Kidney Disease Medical Center, General Hospital, Tianjin Medical University, National Key Clinical Specialty, Tianjin Key Medical Discipline, Tianjin, China; 5 Guangdong Provincial Key Laboratory of Autophagy and Major Chronic Non-communicable Diseases, Key Laboratory of Prevention and Management of Chronic Kidney Disease of Zhanjiang City, Institute of Nephrology, Affiliated Hospital of Guangdong Medical University, Zhanjiang, Guangdong, China

**Keywords:** diabetes, metformin, miR-21, osteogenesis, osteoporosis, sclerostin

## Abstract

**Objective:**

ObjectiveThis study aimed to explore the role of the *miR-21/Mef2c/Sost* pathway in the pathogenesis of diabetic osteoporosis (DOP) and to investigate whether metformin ameliorates DOP by regulating this signaling pathway.

**Methods:**

We compared bone mineral density (BMD), bone turnover biomarkers, and bone miR-21 expression levels between elderly female patients with and without diabetes. Diabetic mice and high-glucose-treated MLO-Y4 osteocytes were used to explore the involvement of the *miR-21/Mef2c/Sost* pathway in DOP. miR-21 mice and MLO-Y4 osteocytes transfected with miR-21 mimics or inhibitors were utilized to verify the regulatory effect of the *Mef2c/Sost* pathway on bone metabolism. Furthermore, we explored the therapeutic effect and underlying mechanism of metformin in improving DOP by targeting the *miR-21/Mef2c/Sost* pathway.

**Results:**

Diabetic patients exhibited decreased bone miR-21 expression and reduced BMD, both of which were positively correlated with glycemic control status. Consistently, miR-21 was significantly downregulated in diabetic mice and high-glucose-cultured MLO-Y4 osteocytes. Both miR-21 and diabetic mice presented elevated protein levels of MEF2C and sclerostin, reduced expression of Cyclin D1 and RUNX2, as well as impaired bone strength and bone quality. As a direct target gene of miR-21, *Mef2c* was upregulated under high-glucose conditions, whereas its expression was reversed by miR-21 overexpression in MLO-Y4 osteocytes. The downstream gene Sost of *Mef2c* showed a consistent expression trend. Mechanically, metformin restored bone miR-21 expression in diabetic mice, increased the protein levels of Cyclin D1 and RUNX2 via inhibiting the *Mef2c/Sost* pathway, and ultimately improved bone strength and bone quality in diabetic mice.

**Conclusion:**

The *miR-21/Mef2c/Sost* pathway critically contributes to DOP pathogenesis. Metformin improves bone health in diabetic mice by restoring miR-21 expression. This study is the first to demonstrate the association between metformin and the *miR-21/Mef2c/Sost* pathway in DOP, highlighting the skeletal protective effect of metformin beyond its glycemic regulation function and providing a novel therapeutic target for DOP treatment.

## 1 Introduction

Recently, the prevalence of diabetes has increased to 11.2% in China (1) and surpassing 20% (2) among older adults. Diabetes leads to a 1.2–3fold increase in fracture risk (3, 4). The mortality rate after a fracture is up to 22.9% in patients with diabetes (5). Further, the fracture in patients with diabetes greatly increases medical expenses (6) and impacts patient life expectancy. Diabetic osteoporosis (DOP) was first proposed by Professor Albright in 1948. However, its pathogenesis remains elusive.

Diabetic osteoporosis is a metabolic bone disease marked by diminished bone strength and compromised bone microarchitecture, manifested as thinned cortical bone and sparse trabecular bone, and it is regarded as a common chronic complication of diabetes (7). Clinically, individuals with type 2 diabetes often present a unique osteoporosis phenotype, characterized by relatively high bone mineral density (BMD) yet an increased risk of fractures, a well-recognised phenomenon termed the “diabetes–bone paradox” (8). Multiple pathogenic factors drive diabetic bone metabolic disorders, including persistent hyperglycemia, insulin deficiency, chronic inflammation, excessive accumulation of advanced glycation end-products (AGEs), and abnormal cross-linking of bone collagen. These factors collectively reduce bone quality and strength, ultimately resulting in the onset of diabetic osteoporosis and elevated susceptibility to fragility fractures (9, 10).

MicroRNAs (miRNAs) are short, noncoding RNAs that promote the inhibition or degradation of target gene mRNA by combining with the complementary site of the 3′-terminal untranslated region (11). They play crucial roles in cell differentiation, proliferation, apoptosis, and tumour development. miR-21 is implicated in glucometabolic or bone metabolic disorders. A decrease in the serum miR-21 level in individuals with postmenopausal osteoporosis positively correlates with bone mineral density (BMD) (12). Additionally, microRNA-21 can promote osteogenic differentiation of bone marrow mesenchymal stem cells (BMSCs) (13). However, the specific mechanism of miR-21 in osteogenesis remains unclear. Myocyte enhancer factor 2c (MEF2C) is a regulatory protein expressed in multiple organs, including the brain, heart, and kidneys (14–16), and suppresses osteogenesis via sclerostin (17). Notably, conditional *Mef2c* knockout mice exhibited improved bone quality and increased BMD (18). We identified *Mef2c* as a target gene of miR-21 by searching the TargetScan database (http://www.targetscan.org/vert_72/) (19) and hypothesized that miR-21 might regulate osteogenesis via the *Mef2c/Sost* pathway and, thus, may be an important therapeutic target for DOP.

Metformin is a first-line oral hypoglycaemic drug with multiple favourable effects, including anti-ageing, antitumour, and anti-polycystic ovarian syndrome activities (20–23). It also has osteoprotective properties and lowers the incidence of DOP (24) and fractures (25). Notably, miR-21 is a downstream target of metformin. Therefore, we hypothesized that metformin might protect bone quality and strength in diabetes via miR-21. We aimed to determine whether miR-21 plays a role in diabetic osteoporosis via the *Mef2c/Sost* pathway and whether metformin intervention improves diabetic bone metabolism via miR-21 regulation.

## 2 Materials and methods

### 2.1 Patient enrolment

We enrolled 245 women aged > 60 years, including 110 who had diabetes for at least 5 years and 135 without the disease. A subgroup of patients (12 with diabetes and nine without) underwent hip replacement surgery. Bone miR-21 expression was analysed in this subgroup. The differences in age, blood pressure, serum creatinine, albumin, blood glucose, HbA1c, bone turnover biomarker levels, calcium/phosphorus metabolism, bone miR-21 expression, and BMD were compared between the diabetic and nondiabetic groups. We examined the correlation between miR-21 expression and blood glucose and HbA1c levels. All individuals provided informed consent, and the study was approved by the Institutional Ethics Committee of the Second Hospital of Tianjin Medical University, Tianjin, China (KY2022K156).

### 2.2 Animal experiments

Six-week-old male homozygous miR-21 knockout mice, generated using clustered regularly interspaced short palindromic repeats-Cas9 technology, were kindly provided by Professor Rongxin Zhang (Basic Medical Research Center, Tianjin Medical University, Tianjin, China). The homozygous genotype of these mice was confirmed via agarose gel electrophoresis (**Supplementary Figure 1**). Wild-type (C57BL/6J) male mice were purchased from Beijing Huafukang Biotechnology Co., Ltd. (Beijing, China).

All experimental protocols were authorized by the Institutional Ethics Committee of Chu Hsien-I Memorial Hospital of Tianjin Medical University, Tianjin, China (DXBYY-IACUC-2022092). The mice were housed in a pathogen-free animal facility at Tianjin Medical University under a 12/12-h light/dark cycle with good ventilation and an ambient temperature between 20 °C and 24 °C. The miR-21 knockout mice (miR-21^-/-^ group, n = 10) were fed a standard diet. The wild-type mice (n = 30) were randomly assigned to three groups: ① normal control (NC, n = 10), ② diabetic (DM, n = 10), and I diabetic with metformin treatment (DM+Met, n = 10).

Mice in the NC group were fed a standard diet, whereas those in the DM group were fed a high-fat diet (60% kcal high-fat diet) and injected with low-dose streptozotocin (30 mg/kg/d in 0.1 mol/L sodium citrate buffer, pH 4.5; Sigma-Aldrich, St. Louis, MO, USA) through the tail vein for 12 weeks. An equivalent volume of citrate buffer (Sigma-Aldrich) was injected into mice in the NC group. A diabetic mouse was defined as an animal with blood glucose ≥ 16.6 mM based on three independent measurements. Diabetic mice treated with 227.5 mg/kg/d metformin (equivalent to 1500 mg/d in humans) via gavage for 10 weeks were identified as the DM+Met group. Mice in the DM group received the same amount of normal saline (China Otsuka Pharmaceutical Co., Ltd., Tianjin, China) via gavage. The animals were euthanized under isoflurane anaesthesia using cervical dislocation.

### 2.3 Haematoxylin and eosin staining

Isolated bone tissues were decalcified using ethylenediaminetetraacetic acid (Boster Biological Technology Co., Ltd., Wuhan, China), paraffin-embedded, and axially sectioned. This was followed by staining with haematoxylin and eosin (H&E) using standard procedures. Finally, the tissue sections were analysed and photographed with an Olympus BX53 Advanced Research Microscope (Tokyo, Japan).

### 2.4 Destructive 3-point bend testing

We assessed the mechanical parametres of the femur using a 3-point bending test. Briefly, the anterior surface of the femur was horizontally positioned on the custom support, facing upward. The midshaft was then vertically loaded at a continuous displacement rate of 0.03 mm/s until a fracture occurred; the span length was 10 mm. Biomechanical variables were calculated based on load-displacement curves. These included the following three indices: ①stiffness: the ratio of the force experienced by the bone and the degree of its deformation that indicates the ability to resist deformation under the external force; ② maximum load: the minimum force required to deform and fracture a bone to evaluate bone fragility; and ③ failure load: the minimum force required to fully fracture a bone.

Microcomputed tomography

Femur specimens obtained from each subject were placed in the scanning chamber of a Siemens microcomputed tomography (micro-CT) imaging system (Siemens, Knoxville, TN, USA). The Inveon Acquisition Workplace scanning software (Siemens) was used to obtain continuous micro-CT images. The scanning voltage, current, and interlayer spacing of the micro-CT system were set to 52 kV, 800 μA, and 19.75 μm, respectively. The 2-D scanned images were reconstructed and analysed in 3-D using COBRA Exxim reconstruction software (EXXIM Computing Corporation, Pleasanton, CA, USA) and Inveon Research Workplace computing software. The bone surface area/bone volume (BSA/BV) and bone volume/total volume (BV/TV) ratios, as well as the trabecular number (Tb. N), trabecular thickness (Tb. Th), trabecular pattern factor (TPF), and trabecular spacing (Tb. Sp) of the specimens were measured.

### 2.5 Cell culture and intervention

MLO-Y4 lineage osteocytes were obtained from Procell Life Science and Technology Co., Ltd. (Wuhan, China). Osteocytes in the NC and high-glucose (HG) groups were cultured for 72 h in the presence of 5.5 and 33.3 mmol/L glucose, respectively. Osteocytes in the HG group were further subjected to metformin treatment at low (10 μM) or high (50 μM) concentrations. Rescue and depletion experiments involved transfecting MLO-Y4 osteocytes with miR-21 mimics and/or inhibitors. Transfection reagents were purchased from Invitrogen (Carlsbad, CA, USA), and transfection efficiency was evaluated at 48 h.

RNA extraction and real-time polymerase chain reaction.

Total RNA was isolated from bones and osteocytes using TRIzol (Ambion, Austin, TX, USA) and reverse-transcribed with a reverse transcription system (Thermo Fisher Scientific). U6 was utilized as a housekeeping gene for miR-21 and mouse GAPDH for others. Real-time quantitative polymerase chain reaction was performed using the SYBR Green polymerase chain reaction kit (Vazyme Biotech Co., Ltd., Nanjing, China). The dissolution curve was generated and analysed using the 2^-ΔΔCq^ method.

### 2.6 Western blot analysis

Total protein was isolated from bones or osteocytes and separated by sodium dodecyl sulphate-polyacrylamide gel electrophoresis. The resolved proteins were transferred onto polyvinylidene fluoride membranes (Millipore, MA, USA), which were then incubated overnight with primary antibodies (anti-MEF2C, 1:1000, BS7160, Bioworld Technology, Co, Ltd., Minneapolis, MN, USA; rabbit polyclonal sclerostin, 1:1000, ab63097, Abcam, Cambridge, UK; mouse monoclonal Cyclin D1, 1:5000, 60186-1-Ig, Miting Biotechnology Co., Ltd., Wuhan, China; rabbit polyclonal RUNX2, 1:1000, PB0171, Boster Biotechnology, Wuhan, China; and rabbit polyclonal GAPDH, 1:1000, AB-P-R 001, Xianzhi Biological Co., Ltd., Hangzhou, China) at 4 °C. Membranes were then incubated with secondary antibodies (horseradish peroxidase-conjugated AffiniPure Goat Anti-rabbit/mouse IgG, Boster Biotechnology, Wuhan, China) and the immunoreactive bands were observed using an enhanced chemiluminescence chromogenic system (Advansta, San Jose, CA, USA). The results were analysed using Bandscan software (Glyko Inc., Novato, CA, USA).

### 2.7 Enzyme-linked immunosorbent assay

The Mouse Sclerostin/SOST PicoKine enzyme-linked immunosorbent assay kit (Boster Biotechnology) was used for enzyme-linked immunosorbent assays. The supernatant in each group was centrifuged at 1500 × *g* for 10 min at 37 °C for direct analysis.

### 2.8 Statistical analysis

SPSS 22.0 software (IBM, Armonk, NY, USA) was used for statistical analyses. The normally distributed data (or log-transformed data) are shown using the mean ± standard deviation (*x̄* ± s). We used the independent samples *t*-test to analyse differences between the two groups and one-way analysis of variance for comparisons among multiple groups; the least significant difference *t*-test was used for pairwise comparisons. Pearson’s correlation analysis was utilized to examine the correlation between different variables. Results with *P* < 0.05 were considered statistically significant. Graphs were plotted using GraphPad 7.0 (GraphPad Inc., San Diego, CA, USA).

## 3 Results

### 3.1 Bone miR-21 expression from patients with diabetes

We did not detect any significant differences between the DM and non-DM groups in index, age, or serum biochemical parametres, such as lipid levels and creatinine. However, fasting blood glucose and HbA1c levels in the DM group were remarkably higher than those in the non-DM group (*P* < 0.05). Patients with diabetes had notably lower BMD according to dual-energy X-ray absorptiometry (*P* < 0.05). In addition, the DM group had significantly higher fasting blood glucose levels and lower serum levels of type I procollagen amino-terminal peptide than the non-DM group (*P* < 0.05) (**Table 1**).

**Table 1:** Comparison of BMD, bone turnover biomarkers, and biochemical indices between 2 groups.

	Non-DM group	DM group	P
Case	135	110	
Age	76.89 ± 12.56	76.73 ± 13.02	0.97
BMI(kg/m^2^)	25.17 ± 4.41	26.14 ± 3.78	0.17
Fasting blood glucose(mmol/L)	5.59 ± 1.55	7.82 ± 3.2^#^	0.00
Femoral neck BMD(g/cm^2^)	0.70 ± 0.14	0.62 ± 0.15^#^	0.00
Total hip BMD(g/cm^2^)	0.86 ± 0.18	0.76 ± 0.16^#^	0.00
Vertebra BMD(g/cm^2^)	0.96 ± 0.23	0.88 ± 0.17^#^	0.02
25-OH VD(ng/ml)	20.61 ± 8.43	19.53 ± 8.59	0.34
β-CTX(ng/ml)	0.41 ± 0.26	0.39 ± 0.26	0.49
PINP*(ng/ml)	59.31 ± 65.03	45.54 ± 33.44^#^	0.04
Ca(mmol/L)	2.32 ± 0.21	2.33 ± 0.16	0.54
P(mmol/L)	1.05 ± 0.21	1.12 ± 0.43	0.14
PTH*(pmol/L)	7.5 ± 7.58	6.64 ± 5.37	1.00
Serum creatinine*(μmol/L)	85.69 ± 32.60	85.24 ± 31.92	0.94
Uric acid(mmol/L)	303 ± 114.04	327.96 ± 108.72	0.09
TG*(mmol/L)	1.44 ± 0.78	1.65 ± 1.31	0.22
TC(mmol/L)	4.77 ± 1.21	4.62 ± 1.20	0.37
HDL(mmol/L)	1.22 ± 0.37	1.20 ± 0.36	0.63
LDL(mmol/L)	2.97 ± 0.96	2.88 ± 0.95	0.71

BMI, body mass index; BMD, bone mineral density; β-CTX, β-C-terminal telopeptide of type I collagen; PINP, type I procollagen N-terminal propeptide; PTH, parathyroid hormone; TG, triglyceride; TC, total cholesterol; HDL, high density lipoprotein-cholesterol; LDL, low density lipoprotein-cholesterol.

*Logarithmic transformation provided a normal distribution of data. ^#^Compared with the non-DM group, P < 0.05.

Further analysis of older patients who underwent hip replacement therapy owing to hip fracture (9 patients in the non-DM group and 12 patients in the DM group) revealed that patients of the DM group exhibited remarkably lower miR-21 expression compared with those in the non-DM group (*P* < 0.05) (**Table 2**). Notably, bone miR-21 expression was negatively correlated with blood glucose and HbA1c levels in the DM group (**Table 3**).

**Table 2:** Comparison of expression of miR-21 in bone tissue, glucose control status, and biochemical indices.

	Non-DM group	DM group	P
Case	9	12	
Age	62.33 ± 3.54	65.33 ± 4.54	0.11
Bone miR21	0.609 ± 0.391	0.083 ± 0.029^#^	0.00
Fasting blood glucose(mmol/L)	5.29 ± 0.5	8.63 ± 2.92^#^	0.00
HbA1c(%)	5.77 ± 0.44	7.43 ± 1.56^#^	0.00
SBP(mmHg)	124.67 ± 7	128.75 ± 6.78	0.20
DBP(mmHg)	78.67 ± 6.14	83.5 ± 4.58	0.07
Albumin(g/L)	40.84 ± 2.70	41.2 ± 3.63	0.80
Serum creatinine(μmol/L)	64.87 ± 10.91	71.83 ± 8.21	0.13

SBP, systolic blood pressure; DBP, diastolic blood pressure.

# Compared with the non-DM group, P < 0.05.

**Table 3:** Correlation analysis of expression of miR-21 in bone tissue and glucose metabolism indices in elderly women with diabetes.

	Correlation coefficient	P
Fasting blood glucose (mmol/L)	-0.459	0.036
HbA1c (%)	-0.449	0.041

### 3.2 Inhibition of osteogenesis via the miR-21/Mef2c/Sost pathway in diabetic mice

**Figure 1:**
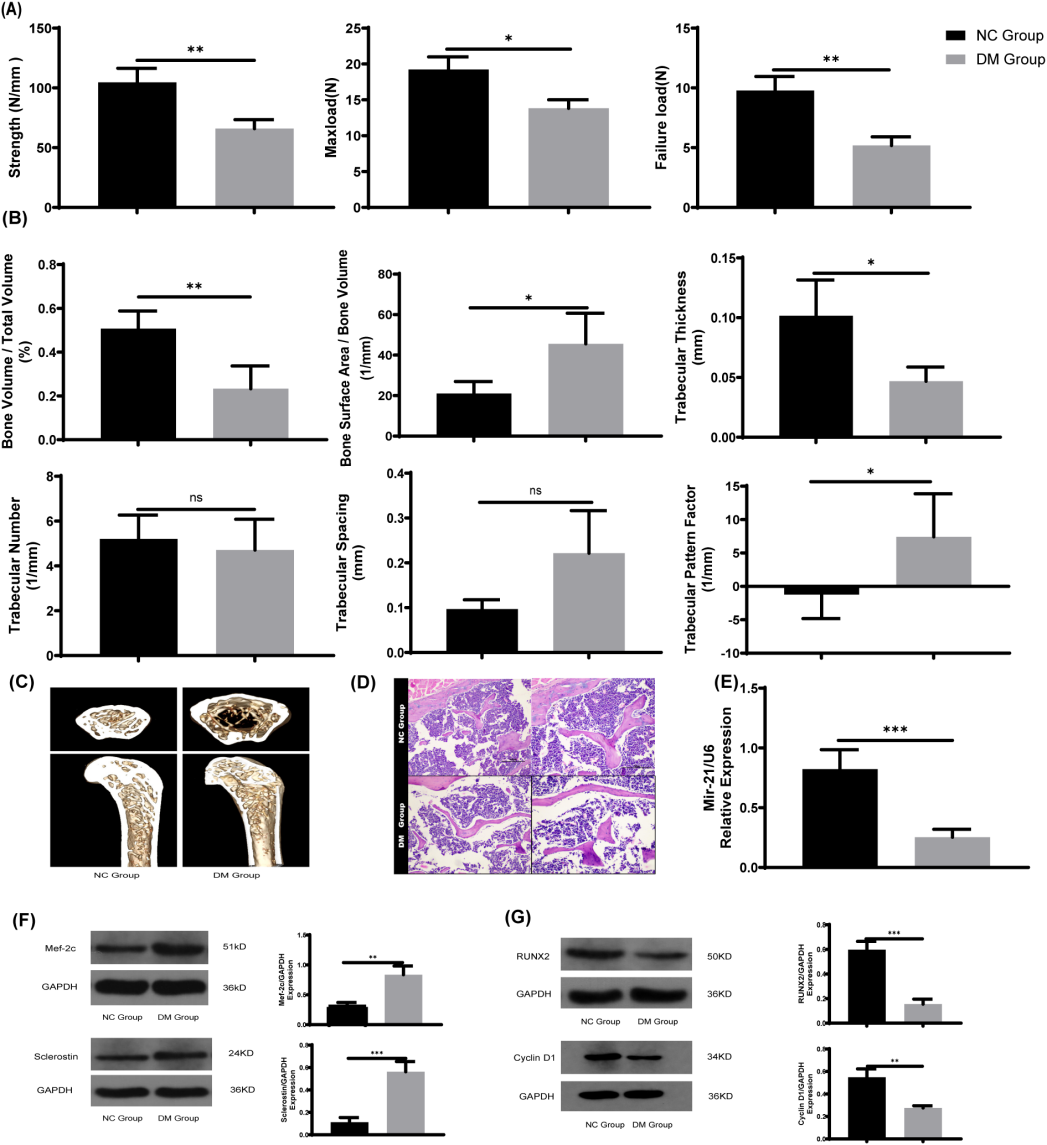
Bone histological characteristics and inhibition of osteogenesis in diabetic mice. **(A)** The 3-point bending test showed that the stiffness, maximum load, and failure load were significantly reduced in diabetic mice. **(B)** microCT analysis of isolated femurs. **(C)** MicroCT image of isolated femurs. **(D)** HE staining indicated that the trabeculae of diabetic mice were sparse and thin. Scale bars: 100 µm (100×) and 50 µm (200×). **(E)** The expression of miR-21 in the bone tissue of diabetic mice was remarkably downregulated. **(F)** The protein levels of MEF2C and sclerostin in the bone tissue of diabetic mice were increased. **(G)** The protein levels of Cyclin D1 and RUNX2 were reduced in the bone tissue of diabetic mice compared with that in controls. ns: P>0.05, *P<0.05, **P<0.005, ***P<0.0005.

No significant differences in body weight were detected between the DM and NC groups. However, the blood glucose levels of mice in the DM group were higher than those in the NC group (*P* < 0.05) (**Supplementary Figures 2A, B**). Additionally, the 3-point bending test results showed that mice in the DM group had a marked decrease in bone stiffness, maximum load, and failure load (*P* < 0.05) (**Figure 1A**). This suggested that diabetes reduced mouse bone strength. Micro-CT analysis demonstrated that mice in the DM group had lower BV/TV and higher BSA/BV ratios (*P* < 0.05). In other words, BMD decreased in diabetic mice. Furthermore, DM mice had a higher TPF (*P* < 0.05), thinner Tb. Th (*P* < 0.05), larger Tb. Sp (*P* > 0.05), and decreased Tb. N (*P* > 0.05) compared with NC mice (**Figures 1B, C**). H&E staining also showed decreased Tb. N and Tb. Th in DM mice (**Figure 1D**). Thus, both micro-CT and H&E staining analyses demonstrated that diabetes degraded bone quality in mice. MiR-21 expression was downregulated in the bone tissue of DM mice (**Figure 1E**), but the protein levels of Mef2c and sclerostin (encoded by *Sost*) were increased (*P* < 0.05). Additionally, cyclin D1 and RUNX2 protein levels (encoded by Wnt pathway response genes) were significantly downregulated (*P* < 0.05) in the DM group compared to that in the NC group (**Figures 1F, G**).

**Figure 2:**
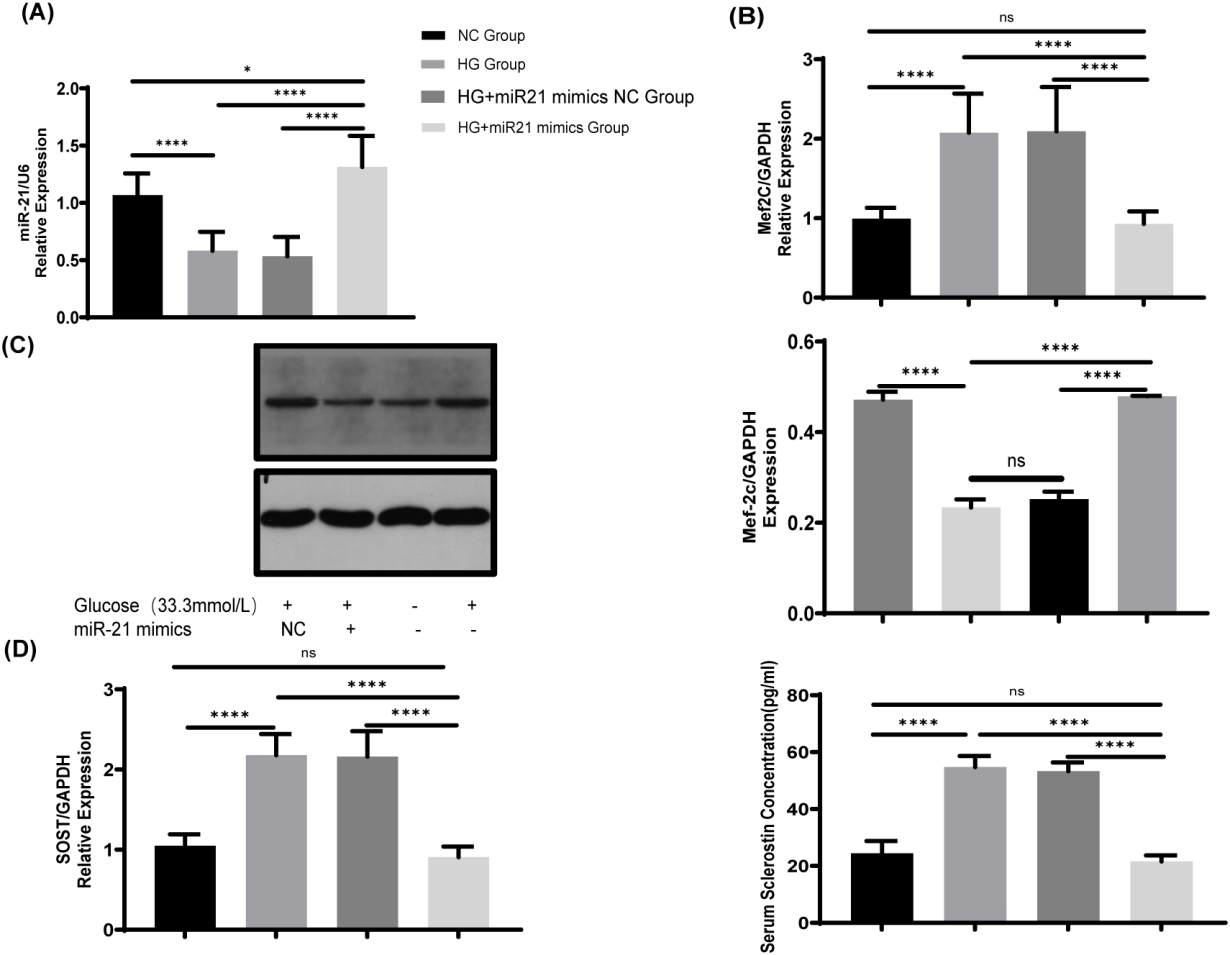
High levels of glucose modulate the miR-21/*Mef2c/Sost* pathway. **(A)** High levels of glucose downregulated the expression of miR-21 in osteocytes, which was rescued by miR-21 mimics. **(B**–**D)** High levels of glucose increased the gene expression and protein levels of MEF2C and Sost/sclerostin. Through add-back experiments, we found that the gene expression and protein levels of MEF2C and Sost/sclerostin were inhibited to some extent by rescuing miR-21 in osteocytes under high glucose conditions. ns: P>0.05, *P<0.05, ****P<0.0001.

MiR-21 suppression was attributed to increased glucose concentration, with the lowest miR-21 expression observed at 33.3 mmol/L glucose. MiR-21 expression in osteocytes from the osmotic pressure control group was comparable with that in the NC osteocyte group (**Supplementary Figure 3**). These results implied that high glucose concentrations inhibited miR-21 expression in osteocytes, while osmotic pressure levels did not. Significantly lower miR-21 expression was observed in MLO-Y4 osteocytes cultured under HG (33.3 mmol/L) for 72 h (*P* < 0.05), while MEF2C and *Sost/*sclerostin gene and protein expression levels were higher. A rescue experiment involving transfecting MLO-Y4 osteocytes with miR-21 mimics in the HG group demonstrated a notable downregulation in the gene expression and protein levels of MEF2C and *Sost/*sclerostin (*P* < 0.05) (**Figure 2**).

### 3.3 Characteristics of bone tissue in miR-21^-/-^ mice

**Figure 3:**
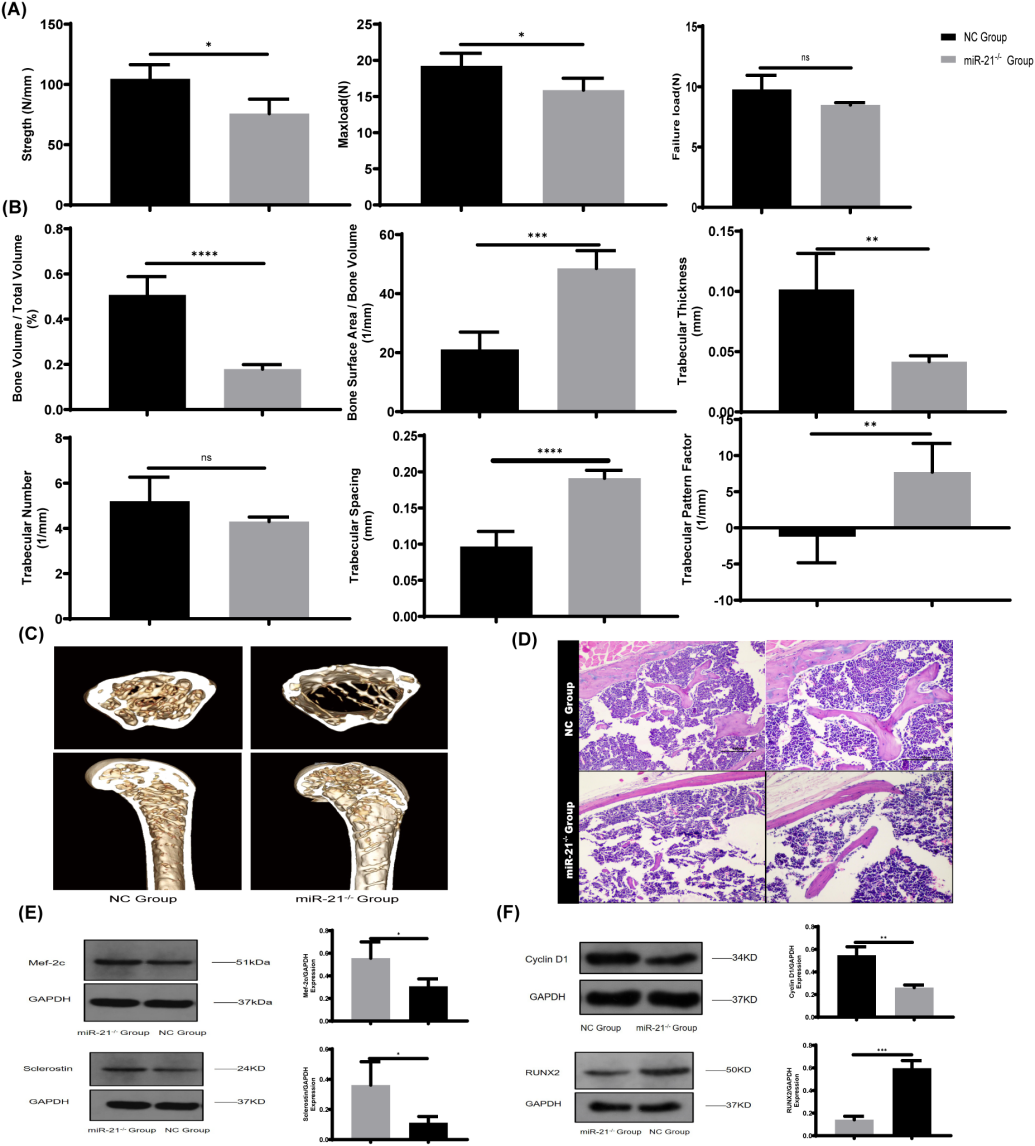
Bone histological characteristics and inhibition of osteogenesis in miR-21^-/-^ mice. **(A)** The 3-point bending test showed that the stiffness and maximum load of bone tissue were significantly decreased in miR-21^-/-^ mice. **(B)** microCT analysis of isolated femurs. **(C)** MicroCT image of isolated femurs. **(D)** HE staining revealed that the bone cortex of miR-21^-/-^ mice was thinner and the trabeculae became sparse. Scale bars: 100 µm (100×) and 50 µm (200×). **(E)** The protein levels of MEF2C and sclerostin were increased in the bone tissue of miR-21^-/-^ mice. **(F)** The protein levels of cyclin D1 and RUNX2 were reduced in the bone tissue of miR-21^-/-^ mice compared with that in controls. ns: P>0.05, *P<0.05, **P<0.005, ***P<0.0005, ****P<0.0001.

No significant differences were observed between miR-21^-/-^ mice and NCs concerning blood glucose and body weight (**Supplementary Figures 2C, D**). miR-21^-/-^ mice showed significantly decreased bone stiffness and maximum load relative to NC mice (*P* < 0.05), with the failure load exhibiting a decreasing trend (*P* > 0.05) (**Figure 3A**). Moreover, micro-CT revealed a decreased BV/TV ratio and an increased BSA/BV ratio in miR-21^-/-^ mice, indicating a lower BMD. miR-21^-/-^ mice exhibited higher TPF, larger Tb. Sp, and lower Tb. Th values (*P* < 0.05), together with a decreasing Tb. N trend (*P* > 0.05). miR-21^-/-^ mice showed trabecular bone thinning and a trabecular degeneration trend (**Figures 3B, C**). MiR-21^-/-^ mice had thinner cortical bone, sparser bone trabecula, and decreased Tb. Th (**Figure 3D**). Bone strength and quality were remarkably reduced in miR-21^-/-^ mice.

**Figure 4:**
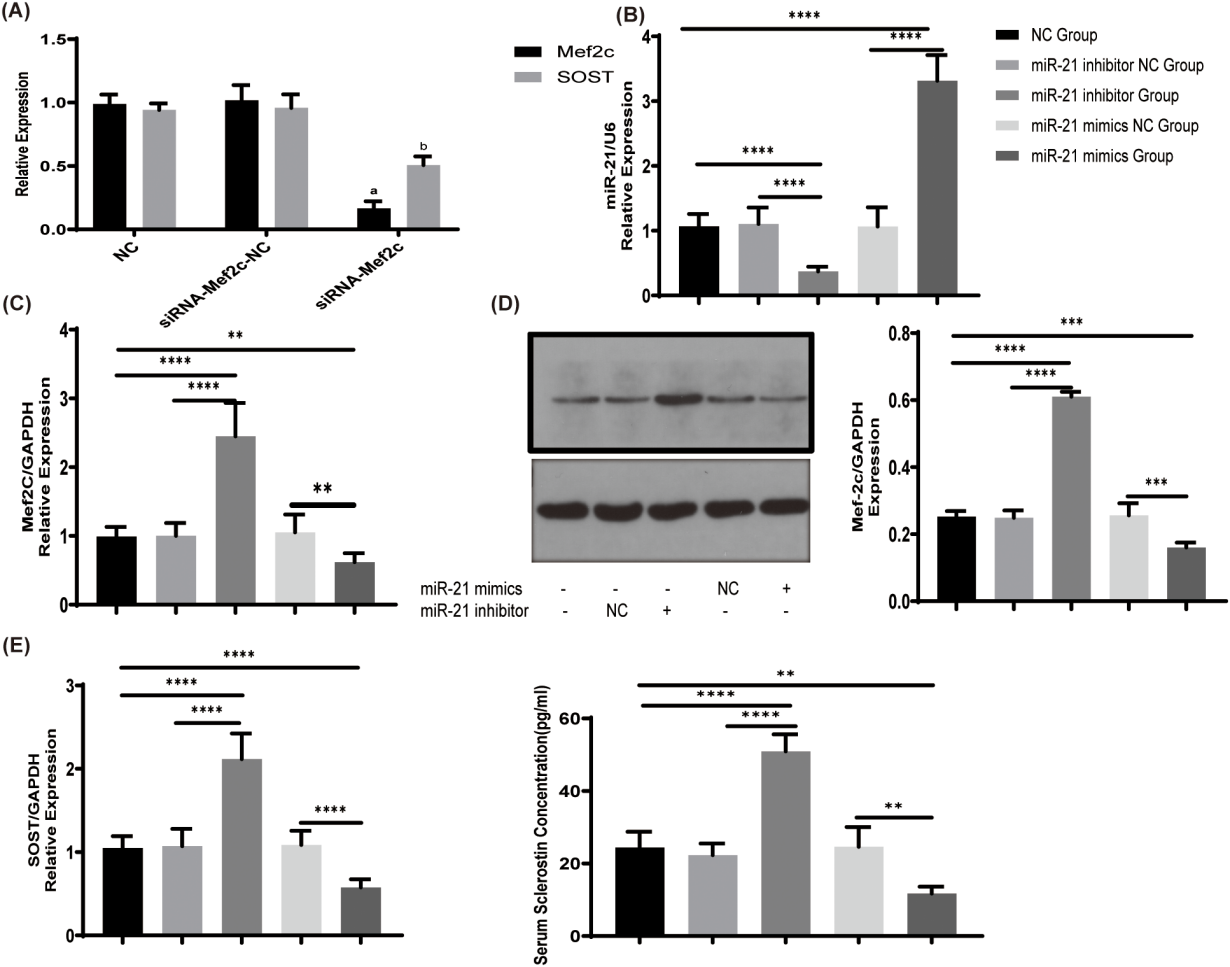
miR-21 targeted the *Mef2c/Sost* pathway in osteocytes. **(A)** *Mef2c* siRNA significantly downregulated the expression of *Sost* mRNA. **(B)** The expression of miR-21 in osteocytes was either upregulated or downregulated following transfection with miR-21 mimics or inhibitors, respectively. **(C–E)** Through depletion and add-back experiments, we found that miR-21 targeted and decrease the gene expression and protein levels of MEF2C and *Sost/*sclerostin in osteocytes **P<0.005, ***P<0.0005, ****P<0.0001..

The protein levels of MEF2C and sclerostin were notably upregulated in the bone tissues of miR-21^-/-^ mice (**Figure 3E**), whereas Cyclin D1 and RUNX2 levels were downregulated (**Figure 3F**). Osteocytes transfected with *Mef2c* siRNA exhibited a marked decrease in *Sost* expression compared with the NC and siRNA NC groups (**Figure 4A**). This confirmed the direct and positive regulatory relationship between *Mef2c* and *Sost*. Results of rescue and depletion experiments showed that miR-21 depletion was associated with the upregulation of MEF2C and *Sost/*sclerostin gene and protein expression levels, while an opposite trend was observed following miR-21 rescue with miR-21 mimics (**Figures 4B–E**).

### 3.4 Metformin protected diabetic mice from the suppression of osteogenesis via the miR-21/Mef2c/Sost pathway

**Figure 5:**
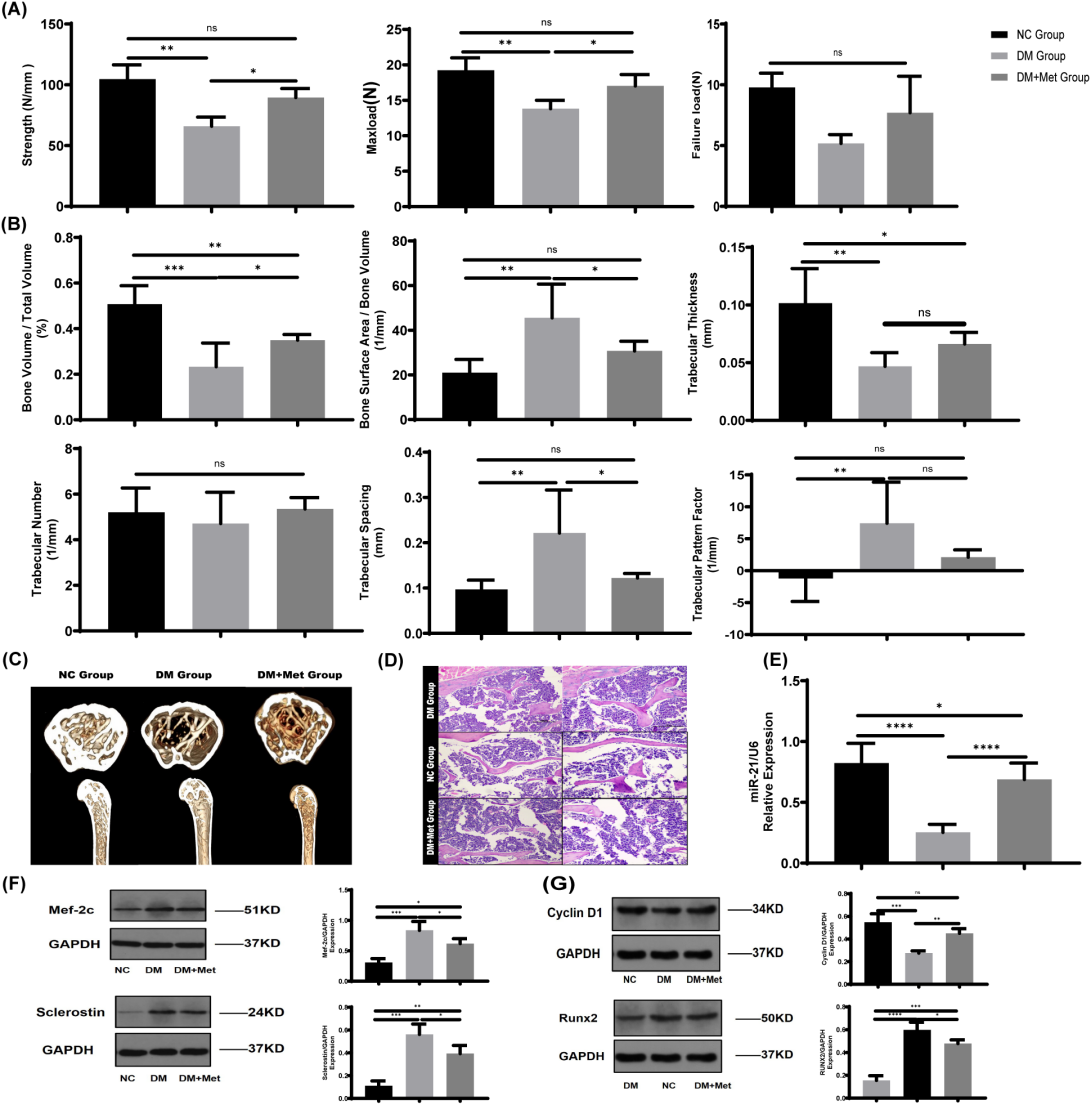
Bone histological characteristics and improvement of diabetes-induced inhibition of osteogenesis in diabetic mice following metformin intervention for 10 weeks. **(A)** The 3-point bending test showed that the stiffness and maximum load were significantly higher in the bone tissue of mice in the DM+MET group than those of diabetic mice, whereas there was no significant difference with those of mice in the NC group. No significant difference was observed in the failure load among the 3 groups. **(B)** microCT analysis of isolated femurs. **(C)** MicroCT image of isolated femurs. **(D)** HE staining showed that the trabecular number was slightly increased in the bone tissue of mice in the DM+MET group compared with that in the DM group. Scale bars: 100 µm (100×) and 50 µm (200×). **(E)** Metformin rescued the expression of miR-21 in the bone tissue of diabetic mice. **(F)** Metformin inhibited the protein levels of MEF2C and sclerostin in the bone tissue of diabetic mice. **(G)** Metformin improved the protein levels of Cyclin D1 and RUNX2 in the bone tissue of diabetic mice. ns: P>0.05, *P<0.05, **P<0.005, ***P<0.0005, ****P<0.0001.

Mice in the DM+Met group had lower blood glucose and body weight compared with those in the DM group (**Supplementary Figures 2E, F**). Moreover, DM+Met mice exhibited remarkably enhanced stiffness and maximal load compared with DM mice based on the 3-point bending test of the femur (*P* < 0.05). Furthermore, stiffness and maximal load had no significant differences between the DM+Met and NC groups. No significant difference in failure load was observed among the three groups (*P* > 0.05) (**Figure 5A**).

Mice in the DM+Met group had higher BV/TV, lower BSA/BV, and lower Tb. Sp values compared with those in the DM group. However, these indices showed no marked differences between the DM+Met and NC mice. Mice in the DM+Met group presented trends of lower TPF and higher Tb. Th values compared to those in DM group, although differences were not statistically significant. Moreover, Tb. N values were comparable among the three groups (**Figures 5B, C**). H&E staining showed that metformin treatment increased Tb. N and Tb. Th in diabetic mice, with the space between the trabecula and the cracks of the trabecula reduced compared with those observed for the DM group (**Figure 5D**).

Bone tissue from metformin-treated mice expressed higher miR-21 levels than DM mice (*P* < 0.05). However, there was no marked difference in miR-21 expression between the DM+Met and NC groups (**Figure 5E**). Furthermore, MEF2C and sclerostin protein levels in bone tissue from DM+Met group mice were downregulated compared with those from DM mice (*P* < 0.05), while Cyclin D1 and RUNX2 protein levels were notably upregulated (*P* < 0.05) (**Figures 5F, G**).

**Figure 6:**
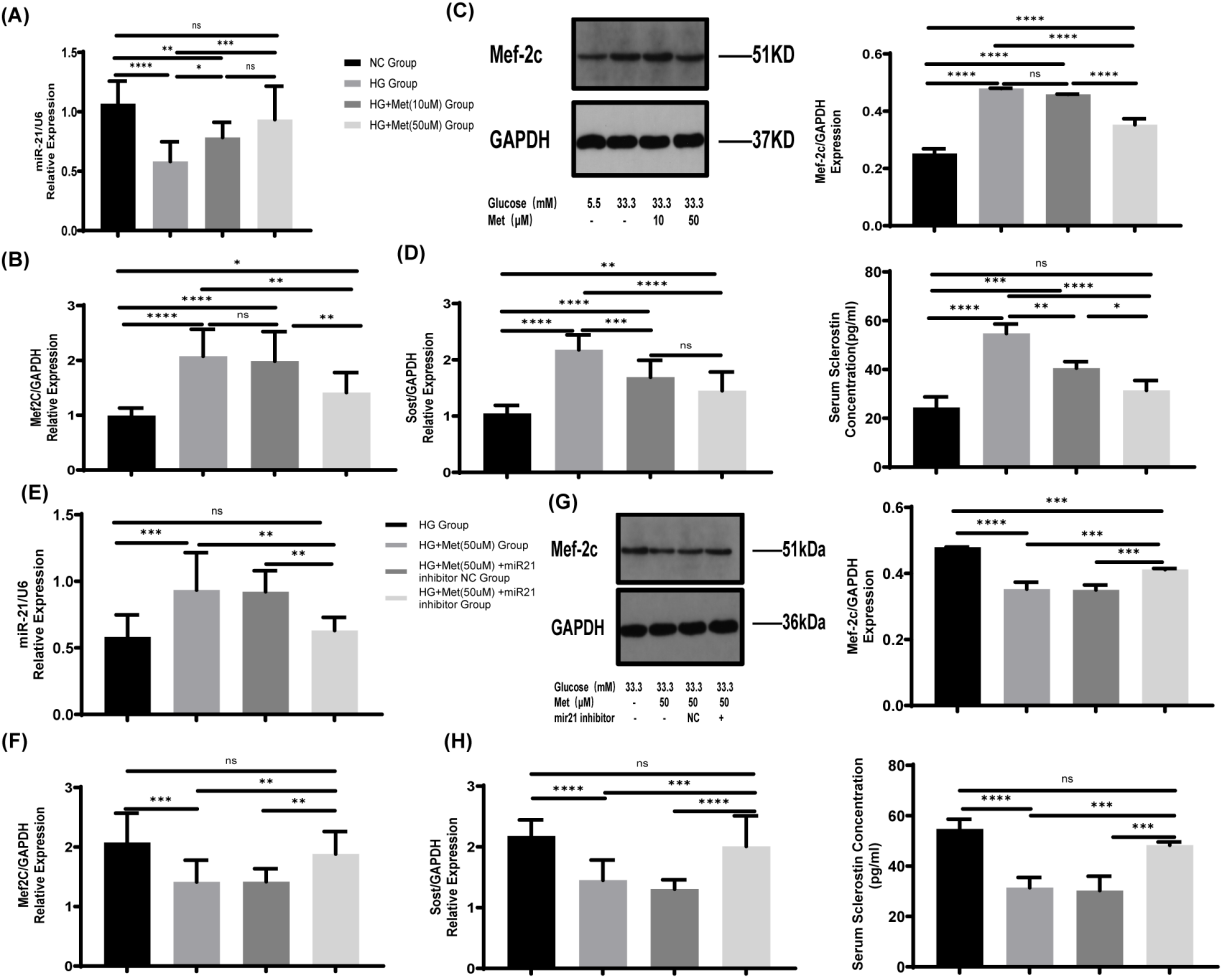
Metformin upregulated the level of miR-21 in osteocytes under high glucose conditions in a concentration-dependent manner, whereas reduced the expression of MEF2C and its downstream *Sost/*sclerostin. **(A–D)** Metformin upregulated the expression of miR-21 in osteocytes under high glucose conditions, whereas repressed that of MEF2C and *Sost/*sclerostin; this effect was greater in the high-dose metformin group. **(E**–**H)** Through add-back experiments, we showed that metformin directly rescued the expression of miR-21 and inhibited the gene expression and protein level of MEF2C and *Sost/*sclerostin in osteocytes under high glucose conditions. ns: P>0.05, *P<0.05, **P<0.005, ***P<0.0005, ****P<0.0001.

In MLO-Y4 osteocytes cultured under HG conditions and subjected to high (50 μM) or low (10 μM) metformin treatment, miR-21 expression increased in the HG+Met (50 μM) and HG+Met (10 μM) groups compared with that in the HG group; however, there were no significant differences in miR-21 expression between the HG+Met (50 μM) and NC groups. The results indicated that metformin upregulated miR-21 expression in a concentration-dependent manner (**Figure 6A**). Notably, metformin decreased the mRNA and protein levels of MEF2C and *Sost/*sclerostin in osteocytes in a concentration-dependent manner (**Figures 6B–D**). Our miR-21 depletion experiment also demonstrated that MEF2C and *Sost/*sclerostin mRNA and protein levels were markedly upregulated in the HG+Met (50 μM) group after osteocytes were transfected with the miR-21 inhibitor relative to those in the HG+Met (50 μM) group (*P* < 0.05) (**Figures 6E–H**).

## 4 Discussion

MiR-21 knockout mice presented lower bone strength and quality. We are the first to observe that miR-21 in the bone tissue of the diabetic population decreased. Downregulation of bone miR-21 expression in mice with diabetes weakened bone strength and quality via the *Mef2c/Sost* pathway. Metformin rescued miR-21 and improved the bone metabolism of diabetic mice.

MiR-21 is involved in proliferation (26), differentiation (27), and metastasis (28). It can also promote wound healing by upregulating collagen synthesis and facilitating re-epithelialization (29). Knockout of miR-21 reduces tumour growth (30) and alleviates acute kidney injury (31). Moreover, miR-21 regulates bone metabolism, although conclusions are inconsistent. Patients with osteoporosis or osteopenia have lower serum miR-21 levels (32), positively associated with BMD. Other studies have reported increased miR-21 in blood (33) and bone tissue (34) of patients with osteoporosis. Furthermore, miR-21 knockout blocks age- and ovariectomy-induced osteopenia in mice by regulating bone resorption (35). Aging accelerates osteocyte apoptosis and enhances osteoclast function by suppressing Cx43 and miR-21 (36). miR-21 deficiency in osteocytes improves bone strength in mice, although contrasting effects of miR-21 have been reported on osteocyte viability and mitochondrial function between the sexes (37).

MiR-21^-/-^ mice exhibited remarkably compromised bone strength and quality. The trabecular bone was sparse and thin in miR-21^-/-^ mice compared to the controls, with considerably decreased Tb. N. These features indicated high fracture risks. Furthermore, miR-21^-/-^ mice showed increased rod-shaped bone trabeculae, suggesting bone microarchitecture deterioration. Our research indicates that miR-21 is an osteoprotective factor that can reduce fracture risk.

*Mef2c*, a validated target gene of miR-21 (19), represses the BMD. *Mef2c* knockout improves bone quality and increases BMD in mice (17). Furthermore, *Mef2c* is the key transcription factor of *Sost*, which encodes sclerostin in mature osteocytes. Our findings confirm the direct and positive regulatory relationship between *Mef2c* and *Sost* in osteocytes. Leupin et al. (38) were the first to propose that treating osteocytes with MEF2 inhibitors and low doses of parathyroid hormone (PTH) downregulates the expression of osteocyte-specific *Sost.* Notably, sclerostin is an important antagonist of the canonical Wnt/β-catenin pathway owing to its binding with low-density lipoprotein receptor-related protein 5/6 receptors (39), inhibiting the cytoplasmic accumulation and nuclear translocation of β-catenin. The consequence is blocking Wnt response gene expression that normally promotes osteogenesis and suppresses osteogenesis.

Our study found that miR-21^-/-^ mice showed increased expression of Mef2c and sclerostin in bone tissue, while key factors involved in osteogenesis (cyclin D1 and RUNX2) were decreased. The *Mef2c/Sost* axis inhibited miR-21^-/-^ mice osteogenesis. *In vitro* experiments with miR-21 mimics or inhibitors confirmed that miR-21 directly suppresses *Mef2c* and *Sost/*sclerostin. Moreover, miR-21 knockout inhibited osteogenesis and reduced bone mass through the *Mef2c/Sost* pathway. This highlights miR-21 as a positive regulator of bone metabolism.

DOP leads to high fracture risks in older individuals (40), decreasing quality of life and increasing healthcare expenditure. Diabetes substantially reduces BMD and inhibits osteogenesis at representative sites in older women. This is consistent with the characteristics of low-turnover osteoporosis. MiR-21 is implicated in diabetes and its complications. However, studies on its exact role in DOP are rare. Our study observed that bone miR-21 expression that was considerably downregulated in older women with diabetes negatively correlated with fasting blood glucose and HbA1c levels. Therefore, we hypothesized that miR-21 plays a crucial regulatory part in DOP pathogenesis.

Bone miR-21 expression in diabetic mice decreased, consistent with findings in diabetic humans and rabbits (41). Diabetes leads to decreased bone strength and quality, increasing the risk of fractures, the most severe complication of osteoporosis. The BMD of diabetic mice decreased, with thinner trabeculae and a larger bone trabecular gap, according to H&E staining. The transformation of the trabecular from a plate-like component to a rod-like morphology is an important feature of bone structural decay. The TPF is used to measure the connectivity and shape of trabecular bone. The TPF value increases during osteoporosis and indicates a change in the trabecular pattern from a plate-like to rod-like morphology. Elevated TPF was observed in diabetic mice, so it was reasonable to assume that miR-21 is a pivotal regulator of DOP.

*In vivo* experiments indicated that low expression of bone miR-21 upregulates MEF2C and sclerostin levels and then suppresses the key proteins of osteogenesis in diabetic mice. *In vitro* experiments showed that HG treatment downregulated miR-21 expression in MLO-Y4 osteocytes in a concentration-dependent manner. The rescue experiments indicated that miR-21 targeted *Mef2c* and *Sost/*sclerostin in osteocytes. This suggested that high glucose levels directly downregulated miR-21 and resulted in the targeted upregulation of MEF2C and sclerostin. In conclusion, diabetes inhibited osteogenesis in mice through the miR-21/*Mef2c/Sost* pathway and considerably compromised bone strength and quality. Thus, miR-21 represents a novel therapeutic target for DOP via the *Mef2c/Sost* pathway.

Metformin promotes osteoblast differentiation by stimulating the secretion of endothelial nitric oxide synthase (42), reducing the RUNX2/PPARγ ratio, and upregulating RAGE (43). It also reduces hyperglycaemia-induced osteoblast apoptosis (44) and inhibits osteoclast differentiation (45), thereby improving bone metabolism. However, the mechanism through which metformin alleviates DOP remains unclear. Our study indicated that metformin upregulated miR-21 expression in the bone tissue of diabetic mice. Mechanical testing further revealed that metformin improved the fracture-prone characteristics of diabetic mice, enhanced bone strength, and ameliorated bone fragility. Concomitantly, it enhanced BMD and improved bone quality while reducing the TPF value of diabetic mice. This suggested an improvement in diabetes-induced morphological trabecular impairment via increased trabecular connectivity. Metformin regulates miR-21 expression in patients with cancer by inhibiting tumour invasiveness (46) via the AMPK/mTOR pathway. Furthermore, its administration lowers fracture risk in diabetic mice (47), accelerates bone healing, and forms more mature bone at the fracture site (48), facilitating increased BMD.

In our study, metformin rescued bone miR-21 expression in diabetic mice, downregulated MEF2C and sclerostin, and promoted osteogenesis, improving bone strength and quality. Other studies also suggest targeting miR-21 or its downstream proteins as potential approaches to ameliorate bone metabolism. Low-dose PTH interventions enhance Wnt expression through the WISP1/MEF2C/SOST pathway, promoting osteogenesis and increasing BMD in osteoporotic mice. Additionally, biocomposites loaded with microRNA-21 promote Bone Marrow Mesenchymal Stem Cells migration and osteogenic differentiation by increasing RUNX2 expression or through the PTEN/PI3K/Akt axis (49).

A limitation of this study is that systemic knockout of miR-21 in mice can induce functional changes in multiple tissues and organs, which may confound bone metabolism. To address this potential confounding factor, we will employ osteocyte-specific miR-21 knockout mice in future studies to exclude influences from non-skeletal tissues and more precisely define the role of miR-21 in regulating bone metabolism.

In conclusion, miR-21 plays an osteoprotective role in diabetes. HG significantly degraded bone strength and quality by inhibiting osteogenesis via the miR-21/*Mef2c/Sost* pathway. Metformin administration improved the prejudicial effects of HG on osteogenesis via miR-21 upregulation in diabetic bone tissues, thereby enhancing bone strength and quality in mice. This reveals the involvement of a new signalling axis in DOP pathogenesis and highlights the therapeutic target of metformin.

## Data Availability

The original contributions presented in the study are included in the article/**Supplementary Material**. Further inquiries can be directed to the corresponding authors.
