# Supplementary material for: Metformin attenuates diabetic osteoporosis via the miR-21 mediated Mef2c/Sost pathway
Source: Front Endocrinol (Lausanne). 2026 Jun 5;17:1841140. doi: 10.3389/fendo.2026.1841140 (PMC13278933; doi:10.3389/fendo.2026.1841140)
Supplement: Supplementary file 1 [file DataSheet1.zip › Frontier-补充图及图例/Supplementary figure legends.docx]

**Supplementary figure legends**

Figure S1 Confirmation of selection of homozygous knockout mice using agarose gel electrophoresis. The presence of a band at 713 bp indicated samples obtained from homozygous miR-21-knockout mice; samples with a band at 782 bp were identified as WT mice; double band indicated heterozygous mice.

Figure S2 Measurements of weight and blood glucose in all groups.

**(A-B)** There were no significant differences in body weight between groups; however, the levels of blood glucose were significantly increased in diabetic mice at the endpoint of the experiment. **(C-D)** Knocking-out miR-21 did not affect the weight and levels of blood glucose of mice. **(E-F)** At the endpoint of the experiment, the weight, and levels of blood glucose of mice in the DM+MET group were significantly lower than those of diabetic mice

Figure S3 Changes in the levels of miR-21 in osteocytes in a time- and glucose-dependent manner.

1. The expression of miR21 was gradually decreased with the prolongation of osteocyte culture under high glucose conditions. **(B)** Compared with the normal glucose group, the expression of miR-21 in MLO-Y4 osteocytes was downregulated with the increase in glucose concentration; however, there was no significant difference in the level of expression of miR-21 in osteocytes cultured under different osmotic pressure conditions.
