# Supplementary figures and images for: Metformin attenuates diabetic osteoporosis via the miR-21 mediated Mef2c/Sost pathway
Source: Front Endocrinol (Lausanne). 2026 Jun 5;17:1841140. doi: 10.3389/fendo.2026.1841140 (PMC13278933; doi:10.3389/fendo.2026.1841140)

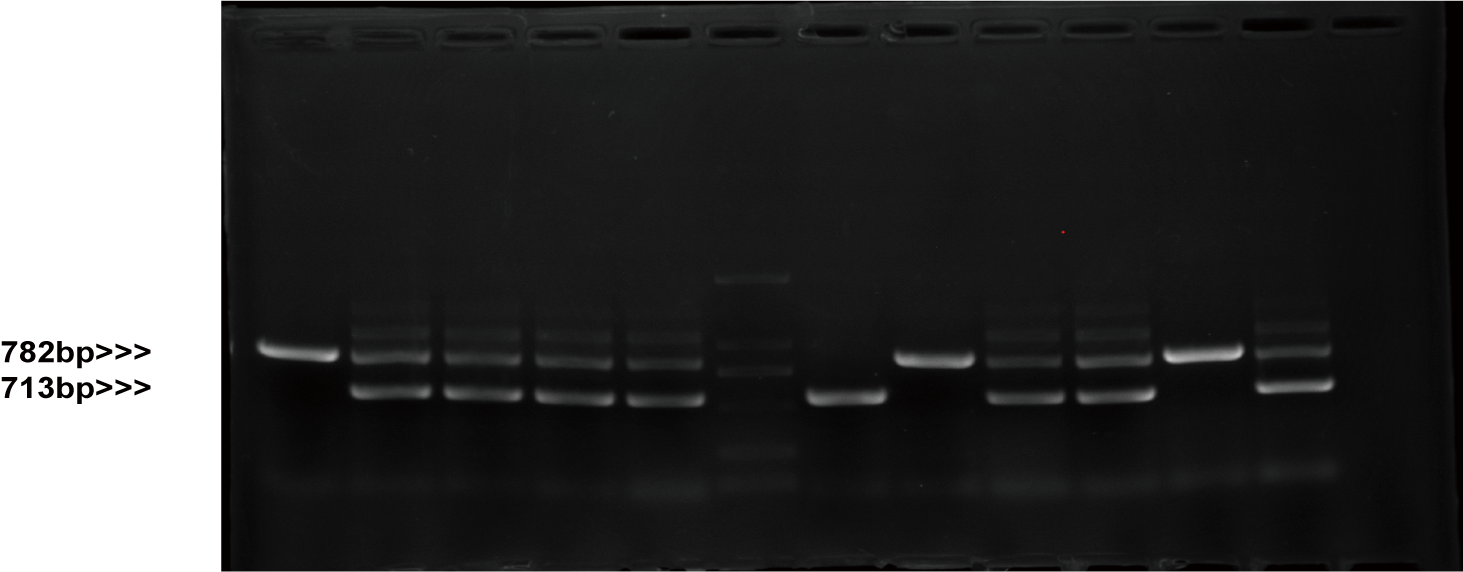

Supplement: Supplementary file 1 [file DataSheet1.zip › Frontier-补充图及图例/S1.tif]

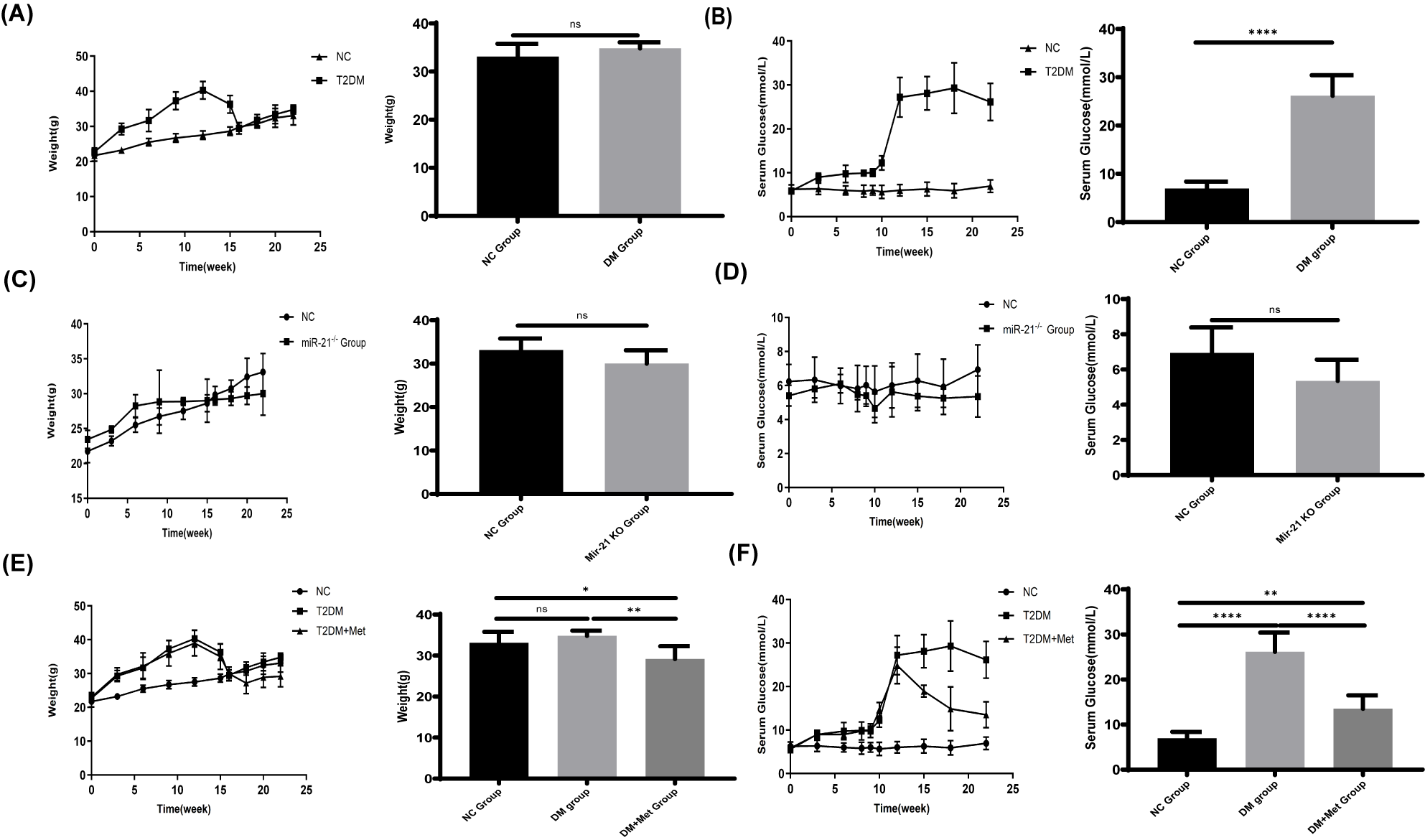

Supplement: Supplementary file 1 [file DataSheet1.zip › Frontier-补充图及图例/S2.tif]

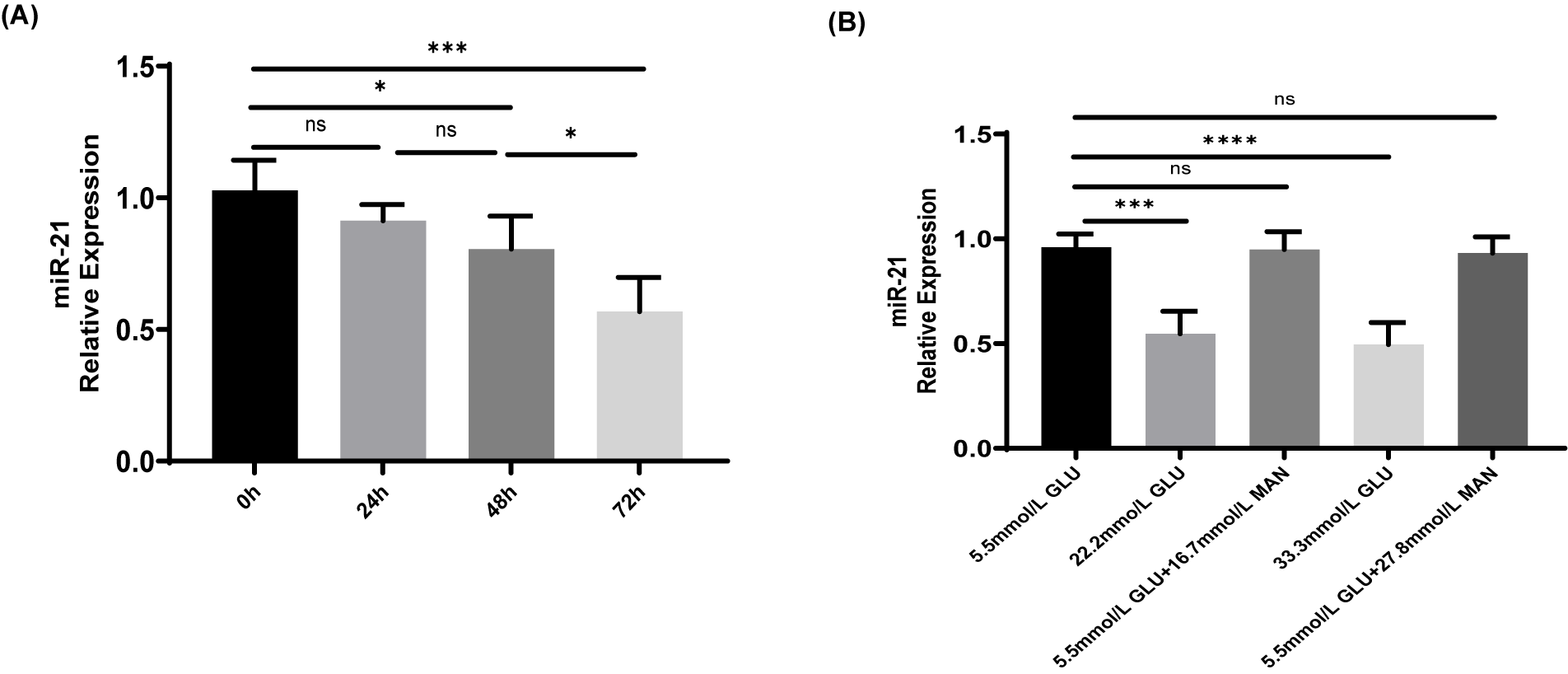

Supplement: Supplementary file 1 [file DataSheet1.zip › Frontier-补充图及图例/S3.tif]
